# Supplementary figures and images for: Vascular Stem Cells and the Role of B-Raf Kinase in Survival, Proliferation, and Apoptosis
Source: Int J Mol Sci. 2023 Apr 19;24(8):7483. doi: 10.3390/ijms24087483 (PMC10138574; doi:10.3390/ijms24087483)

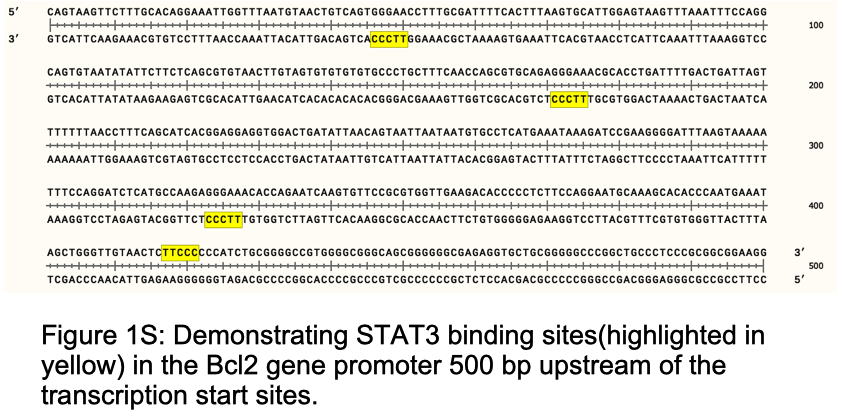

Supplement: Supplementary file 1 [file ijms-24-07483-s001.zip › ijms-2289569-supplementary/Supplemental Figures S1.tif]
